# Dr. William Holden Chace

**Published:** 1908-04

**Authors:** 


Dr. William Holden Chace, of Buffalo, died at his home February 14, 1908. He graduated at the University of Buffalo in 1887.

